# TADF and X-ray Radioluminescence of New Cu(I) Halide Complexes: Different Halide Effects on These Processes

**DOI:** 10.3390/ijms24065145

**Published:** 2023-03-07

**Authors:** Alexander V. Artem’ev, Andrey Yu. Baranov, Alexey S. Berezin, Dmitry V. Stass, Christina Hettstedt, Ul’yana A. Kuzmina, Konstantin Karaghiosoff, Irina Yu. Bagryanskaya

**Affiliations:** 1 Nikolaev Institute of Inorganic Chemistry, 3, Acad. Lavrentiev Ave., Novosibirsk 630090, Russia; 2 Department of Physics, Novosibirsk State University, 2 Pirogova St., Novosibirsk 630090, Russia; 3 Voevodsky Institute of Chemical Kinetics and Combustion SB RAS, 3 Institutskaya St., Novosibirsk 630090, Russia; 4 Department of Chemistry, Ludwig-Maximilian University of Munich, Butenandtstr. 5–13, 81377 Munich, Germany; 5 N. N. Vorozhtsov Novosibirsk Institute of Organic Chemistry, SB RAS, 9, Acad. Lavrentiev Ave., Novosibirsk 630090, Russia

**Keywords:** Cu(I) complexes, thermally activated delayed fluorescence, X-ray radioluminescence, halide effect, phosphorescence

## Abstract

A series of complexes [Cu_2_X_2_(Pic_3_PO)_2_] (X = Cl, Br, I) based on tris(pyridin-2-ylmethyl)phosphine oxide (Pic_3_PO) has been synthesized. At 298 K, these compounds exhibit thermally activated delayed fluorescence (TADF) of ^1^(M+X)LCT type with λ_max_ varying from 485 to 545 nm, and quantum efficiency up to 54%. In the TADF process, the halide effect appears as the emission intensification and bathochromic shift of λ_max_ in the following order X = I < Br < Cl. Upon X-ray irradiation, the title compounds emit radioluminescence, the emission bands of which have the same shape as those at TADF, thereby meaning a similar radiative excited state. By contrast to TADF, the halide effect in the radioluminescence is reversed: its intensity grows in the order X = Cl < Br < I, since heavier atoms absorb X-rays more efficiently. These findings essentially contribute to our knowledge about the halide effect in the photo- and radioluminescent Cu(I) halide emitters.

## 1. Introduction

Metal halide complexes continue to attract ever-increasing attention because of their remarkable functional properties, stability, and facile synthesis [1,2,3,4]. Especially promising are Cu(I) halide complexes featuring intriguing luminescent properties as well as rich structural diversity, various molecular complexity, and robustness [5,6,7,8,9,10,11,12,13,14]. It is well known that upon excitation, the Cu(I) complexes supported by C-, N-, P-, S-, and As-donating ligands can generate the excited states of metal-to-ligand charge transfer type (MLCT) [15,16]. The latter can be radiatively relaxed via phosphorescence or, when the energy gap between S_1_ and T_1_ excited states is relatively small (<1500 cm^−1^), via thermally activated delayed fluorescence (TADF) [16,17,18]. Moreover, the simultaneous appearance of phosphorescence and TADF was demonstrated for some Cu(I) emitters [19,20,21,22]. Because of the great diversity of π-acceptor ligands on the one hand, and various structural motifs of Cu_x_X_y_ units on the other hand, the emission wavelengths of Cu(I)-organic halides can be fine-tuned from deep blue to NIR range. Although emission decay times of Cu(I) complexes do not reveal a clear correlation with the structure, they can also be regulated for specific applications via molecular design approaches [21,23]. Thanks to these advantages, Cu(I) halide complexes are considered promising low-cost TADF and phosphorescent emitters for energy-saving PHOLED and TADF OLEDs, respectively [24,25,26]. Apart from OLED-related applications, Cu(I) halide complexes also attract interest as “smart” materials, since their luminescence often has a reversible response to external stimuli such as temperature, mechanical stress, pressure, and VOCs [6,27]. Most recently, Cu(I) halide hybrids have been proposed as efficient X-ray scintillators generating strong radioluminescence at ambient temperature [28,29,30,31]. Additionally, these materials have found applications in X-Ray imaging [29]. It should be noted that most of the known Cu(I) halide-based X-ray scintillators, i.e., {Cu_4_I_4_} clusters [28,30] and halocuprates (TBA)CuX_2_ (TAB = Bu_4_N) [30], emit phosphorescence. To now, the only Cu(I) scintillator exhibiting TADF is presented by [CuI(PPh_3_)_2_(4-*^t^*BuPy)], reported in 2022 [31]. Although the authors do not claim this complex as a TADF-emitting one [31], similar compounds have previously been proven to be TADF emitters [18].

Meanwhile, despite a plethora of works on Cu(I) halide emitters, the relationships between their structure and photophysical properties are not fully understood. For instance, the impact of halide atoms (Cl, Br, I) on the emission wavelength of Cu(I) halide complexes has been well documented. Generally, a heavier halide induces a hypsochromic shift of the emission bands of the isostructural complexes [21,23,32,33,34,35,36,37,38,39,40,41,42,43], although this dependence is not always explicit [41,44]. The data on the halide effect on the emission type and its intensity are much more scarce [22,42,45]. The influence of a halide atom on the X-ray radioluminescence of Cu(I) emitters is still unexplored, although it has been noted that iodine atoms enhance emission. Again, it is unclear how the variation of halide atoms affects the relationship between efficiencies of photo- and X-ray-excited luminescence. On this account, it is obvious that the elucidation of such relationships is very important for the further development of more efficient Cu(I) emitters for advanced applications.

Herein, we report a series of tris(2-pyridyl)phosphine oxide-supported Cu(I) halide dimers, [Cu_2_X_2_(Pic_3_PO)_2_] (X = Cl, Br, I), showing efficient TADF and X-ray radioluminescence at ambient temperature. It is noteworthy that the TADF intensity of these compounds enhances in the order I < Br < Cl, whilst the radioluminescence improves in reverse order. To our knowledge, similar effects have not been previously described. The molecular/electronic structures as well as photophysical and radioluminescence properties of the designed compounds are also discussed.

## 2. Results and Discussion

### 2.1. Synthesis and Characterization

**Scheme 1:**
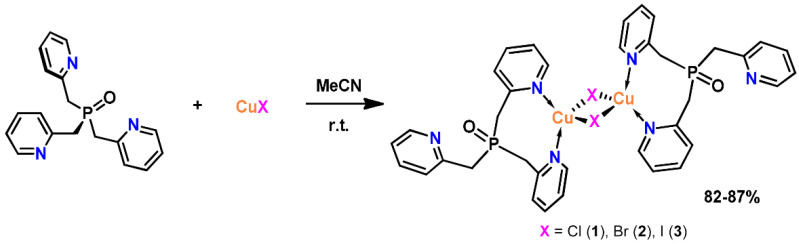
Reaction of Pic_3_PO with Cu(I) halides.

Previously, two research groups [43,46] independently reported the formation of scorpionate complexes by the reaction of tris(2-pyridyl)phosphine oxide (Py_3_PO) with Cu(I) halides. By contrast, herein we have found that tris(pyridin-2-ylmethyl)phosphine oxide (Pic_3_PO), a more flexible ligand, interacts with CuCl, CuBr, or CuI to form dinuclear complexes [Cu_2_X_2_(Pic_3_PO)_2_] (**1**–**3**), in which the ligands feature N,N’-chelating coordination (Scheme 1). The reaction occurs at the equimolar ratio of the reactants in acetonitrile (r.t., 5 h) to afford the solvated complexes [Cu_2_X_2_(Pic_3_PO)_2_]·2MeCN in 82–87% yields. Note that varying the CuX/Pic_3_PO molar ratios does not result in other products, apart from the above ones. The observed difference in the reactivity of Pic_3_PO and Py_3_PO ligands toward Cu(I) halides can be explained by the higher flexibility of Pic_3_PO; thereby the formation of dimeric complexes, rather than scorpionate ones (as in the case of Py_3_PO [43,46]), becomes more favorable.

The phase purity of the prepared products has been proved by both powder X-ray diffraction (Figure S4) and microanalysis data, which show that the experimental results agree well the calculation data. Mid-IR spectra of solvates [Cu_2_X_2_(Pic_3_PO)_2_]·2MeCN inter alia contain specific bands at 2445–2448 cm^−1^, belonging to ν_C≡N_ stretching vibrations of lattice MeCN molecules (Figure S6). Overall, IR spectra of **1**–**3** are very similar to that of free Pic_3_PO [47], except for the bands associated with ν_C=C_ and ν_C=N_ vibrations of pyridine rings. In complexes **1**–**3**, these bands are shifted in higher wavenumbers by 2–5 cm^−1^ compared to those of Pic_3_PO [47], thus confirming the coordination of the ligand to Cu(I).

According to TGA and DTA data (Figure S5), losing the MeCN molecules of [Cu_2_X_2_(Pic_3_PO)_2_]·2MeCN occurs in the range of 50–150 °C. The desolvated complexes **1**–**3** themselves start to decompose at about 160 °C. Through this method, non-solvated complex **1** has also been obtained, the photophysics of which were studied in comparison with **1**·2MeCN (vide infra).

**Figure 1:**
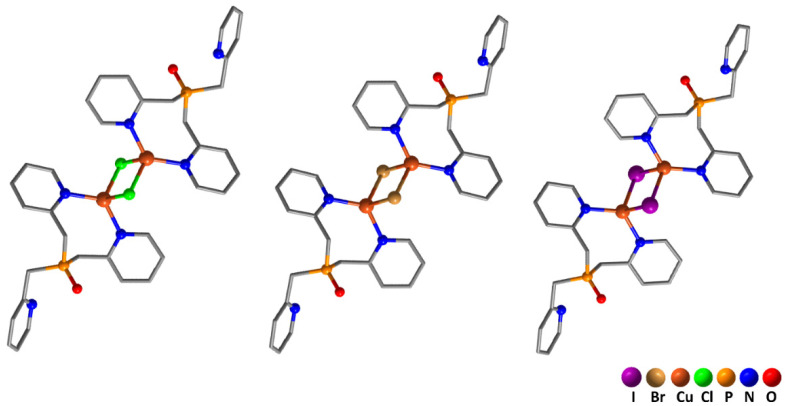
Structures of complexes **1**·2MeCN, **2**·2MeCN, and **3**·2MeCN (hydrogen atoms and solvate molecules are omitted for clarity).

The molecular structures of complexes **1**–**3**, established for their acetonitrile solvates [Cu_2_X_2_(Pic_3_PO)_2_]·2MeCN, are shown in Figure 1 (for more details, see Table S1 and Figures S1–S3). The selected bond lengths are summarized in Table 1. The [Cu_2_X_2_(Pic_3_PO)_2_] molecules have a symmetric structure with the inversion center lying between two Cu atoms. Each metal atom of the rhomboid Cu_2_X_2_ unit is N,N’-chelated by the Pic_3_PO ligand, thereby adopting a distorted tetrahedral {Cu@N_2_X_2_} arrangement with a tetrahedricity τ_4_ index of about 0.85. The intramolecular Cu···Cu distances are elongated in the series **1**·2MeCN (3.122 Å) < **2**·2MeCN (3.224 Å) < **3**·2MeCN (3.274 Å) due to the increase in the halide atom size (Cl < Br < I). The comparison of the Cu···Cu separations with twice Bondi’s van der Waals radius of Cu (2.80 Å) allows metallophilic interactions to be ruled out in these complexes. The Cu–N and Cu–X bond lengths are comparable with those of the related Cu(I) complexes, and C–N, C–C, C–P, and P–O bond lengths in the ligand scaffold correlate well with those in the free ligand [47,48]. The dihedral angles between averaged planes of the coordinated pyridine rings of **1**–**3** are 42.5°, 42.3°, and 44.1°, respectively. In crystal packing, the [Cu_2_X_2_(Pic_3_PO)_2_] molecules form a 3D supramolecular structure by mean of weak C–H···O/N/X contacts as well as π···π stacking interactions between coordinated pyridine rings. The distance between two parallel pyridine rings of **1** is just 3.282 Å (*cf.* 3.314 Å in **2**, 3.462 Å in **3**), while the displacement angle β_Py–Py_ is ~30.9° (*cf.* 28.9° in **2**, 26.6° in **3**). Therefore, the π···π interactions are strong in **1**·2MeCN, are apparent in **2**·2MeCN, and are almost absent in **3**·2MeCN. Note that the lattice MeCN molecules of (**1**–**3**)·2MeCN do not form short intramolecular contacts in the crystals. This is consistent with the fact that desolvation of these compounds begins already at 50 °C (vide supra). It should be remarked that the metal complexes bearing Pic_3_PO ligands were unknown until now.

### 2.2. Theoretical Consideration

**Figure 2:**
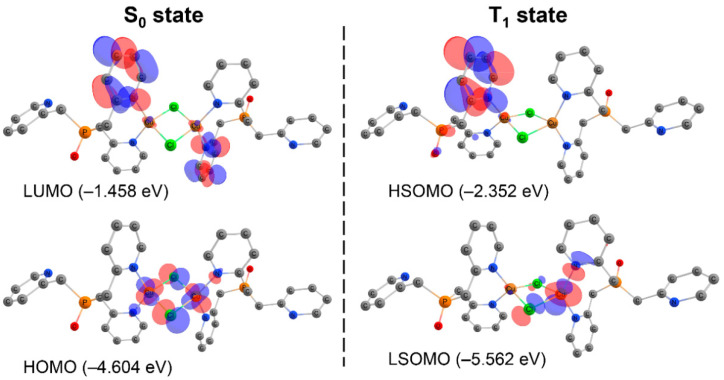
Frontier orbitals of ground (S_0_) and lowest triplet (T_1_) excited states of **1** calculated at B3LYP/def2TZVP level (isosurface = 0.04).

To elucidate the electronic structure of complexes **1**–**3**, we have performed DFT calculations for chloride **1** as a representative example. Its structure is optimized in the ground (S_0_) and lowest triplet (T_1_) excited state at the B3LYP/def2TZVP level of theory (more details are given in §6, ESI). Note that the B3LYP functional and def2TZVP basis set are widely used for the calculation of emissive Cu(I) complexes, providing reasonable agreement with the experimental results [16,17,20,21,22,23,33,35,37,39,43,44]. It is revealed that the HOMO and nearby HOMO-n (n = 1–11) are mainly located on the Cu_2_Cl_2_ unit, while LUMO and nearby LUMO+n are distributed over the pyridine rings. The pattern is observed for the lowest and highest single occupied molecular orbitals (LSOMO and HSOMO) of the T_1_ state of **1** (Figure 2). Such distribution of the frontier orbitals suggests that the lowest excited states of **1** should be of metal+halide-to-ligand character, (M+X)LCT, which is very specific for Cu(I) halide emitters [15,16]. The fact that the HOMO and LUMO of **1** are separated well in space points to a small energy gap between S_1_ and T_1_ excited states that, in turn, gives a reason to expect a TADF manifestation at ambient temperature. Upon S_0_ → T_1_ excitation, the structure of 1 undergoes significant geometric distortions, which mainly appear in pyridyl rings and Cu_2_Cl_2_ units (Table S2). The latter, being planar in the S_0_ state, becomes a butterfly-shaped one with an angle between two CuCl_2_ planes of 159.08° (Table S2). Note that the S_0_ → T_1_ excitation brings about elongation of the Cu∙∙∙Cu distance from 3.039 to 3.241 Å, which points to the absence of metallophilic interactions in the excited state. Time-dependent DFT calculations also predict the (M+X)LCT character of the low-energy electronic transitions in **1** (Table S3, Figure S13), thereby confirming the above assignment. Considering the similarity of absorption spectra of **1**–**3** and the literature data on Cu(I) halide complexes, we believe that the predictions made for **1** are valid for compounds **2** and **3** too.

### 2.3. TADF Properties

**Figure 3:**
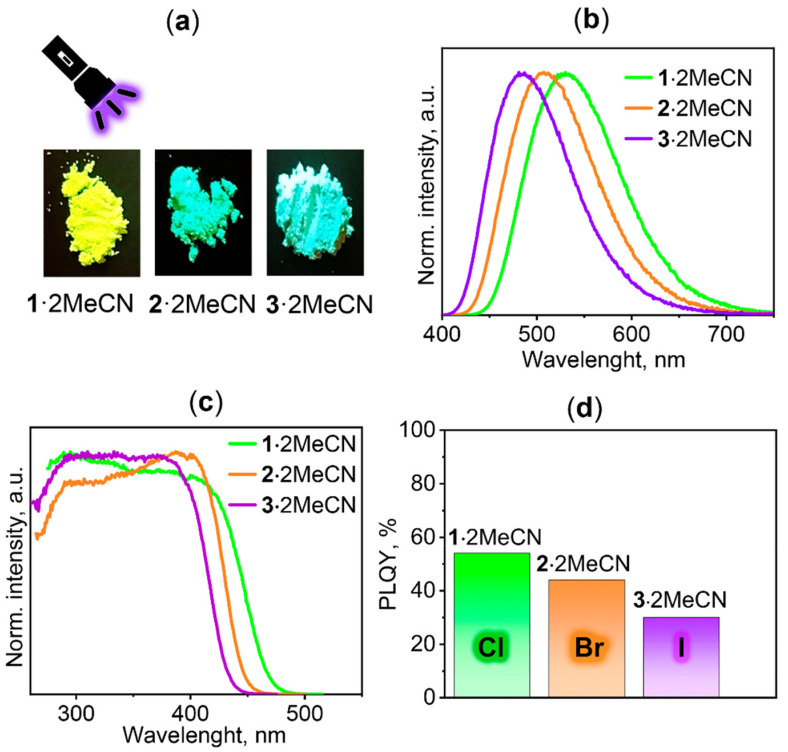
Room temperature photoluminescence of **1**·2MeCN, **2**·2MeCN, and **3**·2MeCN: (**a**) photo of UV irradiated samples; (**b**) emission spectra (λ_ex_ = 390 nm); (**c**) excitation spectra; (**d**) absolute PL quantum yields.

UV irradiation of solid samples of (**1**–**3**)·2MeCN at 298 K results in the appearance of moderate to strong photoluminescence (PL) in the cyan-to-green region (Figure 3a). The emission and excitation spectra are presented in Figure 3b,c, and the corresponding photophysical data are given in Table 2. The emission spectra show broad bands typical for Cu(I) complexes with charge-transfer emission. The emission maxima (λ_max_) of (**1**–**3**)·2MeCN correlate well with the observed PL color (Figure 3a). As seen from Table 2, in the series **1**·2MeCN—**2**·2MeCN—**3**·2MeCN, the λ_max_ shifts in the shorter wavelength domain due to the weakening of ligand field strength of halides in the same order (Cl > Br > I). Previously, this effect was established for many Cu(I) halide emitters. The PL quantum yields (Φ_PL_) vary from 30% (**3**·2MeCN) to 54% (**1**·2MeCN), and the PL lifetimes lie in the microsecond range (4.3–9.0 µs), implying TADF or phosphorescence origin of the luminescence. Thus, the PL quantum yield decreases in the following order: chloride **1**·2MeCN > bromide **2**·2MeCN > iodide **3**·2MeCN (Figure 3d). The excitation spectra (Figure 3c) reveal featureless curves with a maximum intensity below 430–450 nm; the excitation edge falls close to 470 nm for **1**·2MeCN, 445 nm for **2**·2MeCN, and 435 nm for **3**·2MeCN. Overall, the excitation spectra (Figure 3c and Figures S9–S12) closely resemble the absorption profiles (Figure S7). The observed overlapping of the excitation and emission curves implies a significant spin-orbital coupling effect in the title compounds.

The complex **1**·2MeCN demonstrate a solvatochromic luminescence. Thus, its desolvation into **1** brings about red shifting of λ_max_ by 15 nm, dropping the quantum yield to 35%, and elongation of decay time to 5.2 µs (Table 1, Figure S8). This effect can be well rationalized by the formation of voids in solid **1** from leaving lattice MeCN molecules [49]. As a consequence, the matrix environment of molecules of **1** becomes less rigid, which enhances excitation-driven geometric distortions (proved by our DFT calculations, see Table S2). On this account, the Franck–Condon factors between vibrational modes of electronic ground and excited states become higher [50,51], thereby increasing non-radiative deactivation processes, and thus decreasing the emission quantum yield.

**Figure 4:**
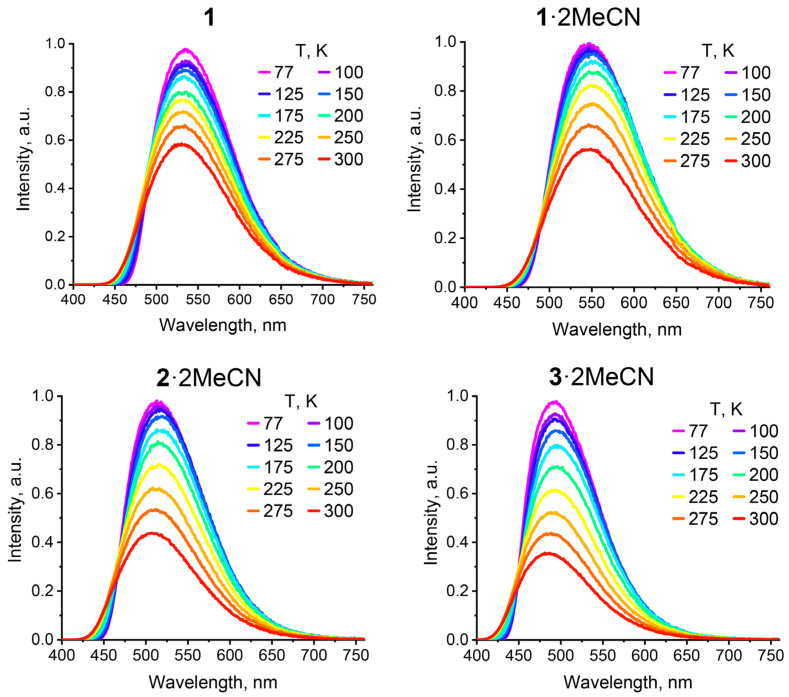
Temperature-dependent emission spectra of solid **1**, **1**·2MeCN, **2**·2MeCN, and **3**·2MeCN, recorded at λ_ex_ = 390 nm.

**Figure 5:**
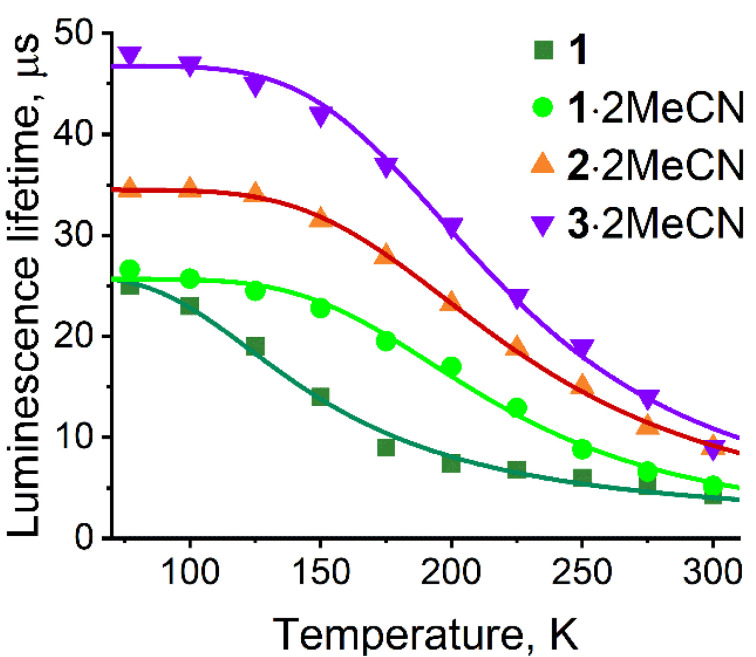
Emission lifetimes of **1** and **1**·2MeCN, **2**·2MeCN, and **3**·2MeCN versus temperature (λ_ex_ = 390 nm). The fitting curves are derived from the equation proposed for the TADF model.

A gradual decrease of temperature from 300 to 77 K intensifies the emission bands of (**1**–**3**)·2MeCN and shifts their maxima and right flanks to the longer wavelength region by 5–10 nm (Figure 4). At that, the emission lifetimes noticeably increase, and their temperature dependence plots τ(T) (Figure 5) suggest manifestation of TADF at ambient temperature. For solvates (**1**–**3**)·2MeCN, the τ(T) curves reach the low-temperature plateau (pure phosphorescence regime) at about 100 K, whereas the τ(T) curves of **1** do not reach this plateau even at 77 K. By contrast, the τ(T) curve of **1**·2MeCN likely reaches a high-temperature plateau (pure TADF regime) at ambient temperature. The curves of (**1**–**3**)·2MeCN obviously continue to decrease at this temperature, meaning an admixture of phosphorescence to the major (TADF) emission channel [19,21,22,45]. Fitting the τ(T) datasets (Figure 5) with a Boltzmann type equation proposed for the TADF model [17] allows the energy gap between T_1_ and S_1_ excited states to be roughly estimated as ∆E(S_1_–T_1_) = 391, 762, 734, and 765 cm^−1^ for **1**, **1**·2MeCN, **2**·2MeCN, and **3**·2MeCN, respectively. The estimated ∆E(S_1_–T_1_) magnitudes being below 1500 cm^−1^ enables the reversible intersystem crossing (T_1_ → S_1_) at ambient temperature, which is required for the appearance of TADF.

### 2.4. X-ray Radioluminescence

**Figure 6:**
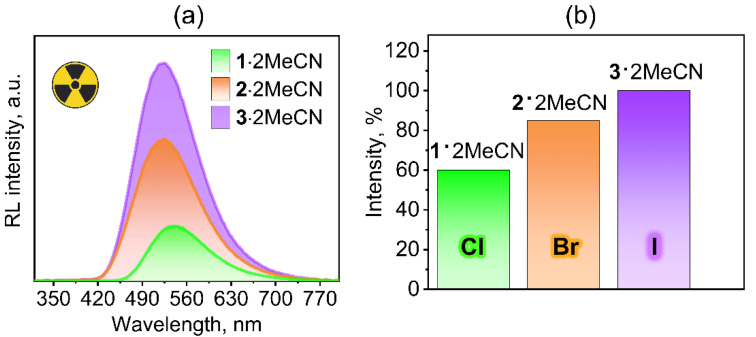
(**a**) X-ray radioluminescence spectra of solid **1**·2MeCN, **2**·2MeCN, and **3**·2MeCN at 298 K; (**b**) Relative intensities of measured radioluminescence.

At ambient temperature, solid samples of (**1**–**3**)·2MeCN display quite strong X-ray radioluminescence (RL). The recorded RL spectra (Figure 6a) match well with the corresponding PL bands (Figure 3b), thus confirming that the same excited states are active in both processes. Overall, the similarity of PL and RL spectra is typical for metal–organic phosphors. The relative RL intensities, measured for thin samples of similar thickness and normalized by sample amount, increase in the order **1**·2MeCN (60%) < **2**·2MeCN (85%) < **3**·2MeCN (taken as 100%) (Figure 6b). It is seen that iodide complex exhibits stronger RL than bromide, let alone the chloride one, which reverses the trend observed for PLQY. The order of RL increase should be attributed to more efficient X-ray absorption by higher-Z atoms [I(Z = 53) > Br(Z = 35) > Cl(Z = 17)] in otherwise isostructural compounds, leading to more efficient harvesting of the exciting high-energy radiation and creation of a higher emission of electronically excited states overcompensating for the decreasing emission efficiency for heavier halogens. Furthermore, the compounds studied display a linear response to X-ray dose rate (Figure S15), though they are sensitive to X-ray irradiation and show noticeable degradation during ca. 1 h of experiment (Figure S14).

## 3. Materials and Methods

### 3.1. General

**Materials and methods.** CuI (99%, Sigma, St. Louis, MO, USA) and MeCN (HPLC grade, Cryochrom, St. Petersburg, Russia) were used as purchased. CuBr was freshly synthesized by treatment of CuBr_2_ with Cu powder in MeCN solution. CuCl (≥99%, Sigma) was additionally purified prior to use by subsequent washing with HCl. Tris(pyridin-2-ylmethyl)phosphine oxide was prepared following the known procedure [47]. XRPD analyses were performed on a Shimadzu XRD-7000 diffractometer (Cu-Kα radiation, Ni—filter, 3–35° 2θ range, 0.03° 2θ step, 5 s per point). FT-IR spectra were collected on a Bruker Vertex 80 spectrometer. The microanalyses were carried out on a MICRO cube analyzer. Thermogravimetric analyses were performed in a closed Al_2_O_3_ pan under helium flow at 10 °C/min^−1^ heating rate using a Netzsch STA 449 F1 Jupiter STA.

The solid-state reflectance spectra were recorded on a Shimadzu UV-3101 spectrophotometer. Samples were prepared by thorough grinding of a mixture of a complex (*ca.* 5 mol%) with BaSO4. The reflectance data were converted into a spectrum by applying a Kubelka–Munk function using BaSO_4_ as a standard.

Emission and excitation spectra were recorded on a Fluorolog 3 spectrometer (Horiba Jobin Yvon) equipped with a cooled PC177CE-010 photon detection module and an R2658 photomultiplier. The absolute PLQYs were determined at 298 K using a Fluorolog 3 Quanta-phi integrating sphere. Temperature-dependent excitation and emission spectra, as well as emission decays, were recorded using an Optistat DN optical cryostat (Oxford Instruments) integrated with the above spectrometer.

X-ray radioluminescence (RL) spectra were recorded on a home-built spectrometer [52] following the earlier developed protocol for powder samples as described in more detail in ESI. The setup includes a CW X-ray tube 2,5BSV-27-Mo with a 40 kV DC anode voltage and an objective-based optical imaging system followed by a grating monochromator and a Hamamatsu H10493-012 PMT photosensor module, and is optimized for measuring naked powder samples in air at ambient conditions.

### 3.2. Synthesis and Characterization Data

**General procedure for the preparation of [Cu_2_(Pic_3_PO)_2_X_2_]·2MeCN:** To a stirred solution of corresponding copper(I) halide (0.155 mmol) in acetonitrile (2.5 mL), tris(pyridin-2-ylmethyl)phosphine oxide (50 mg, 0.155 mmol) was added. The reaction mixture was stirred at ambient temperature under argon for 5 h. The precipitated powder was centrifuged, washed with small amount of acetonitrile, and dried in vacuum.

**[Cu_2_(Pic_3_PO)_2_Cl_2_]·2MeCN** (**1**·2MeCN). Off-white powder. Yield: 125 mg (87%). *Anal*. Calc. for C_40_H_42_Cl_2_Cu_2_N_8_O_2_P_2_: C, 51.8; H, 4.6; N, 12.1%. Found: C, 52.0; H, 4.5; N 12.2%. FT-IR (KBr, cm^−1^): 407 (m), 420 (m), 498 (s), 729 (m), 752 (m), 764 (s), 795 (m), 812 (m), 831 (m), 854 (s), 868 (s), 995 (w), 1051 (m), 1138 (m), 1148 (m), 1175 (vs), 1204 (m), 1217 (s), 1258 (s), 1310 (m), 1395 (m), 1410 (m), 1435 (vs), 1476 (vs), 1566 (m), 1597 (s), 2249 (vw), 2887 (w), 2922 (m), 3019 (w), 3053 (w), 3069 (w).

**[Cu_2_(Pic_3_PO)_2_Br_2_]·2MeCN** (**2**·2MeCN). Off-white powder. Yield: 134 mg (85%). *Anal*. Calc. for C_40_H_42_Br_2_Cu_2_N_8_O_2_P_2_: C, 47.3; H, 4.2; N, 11.0%. Found: C, 47.1; H, 4.2; N, 11.1. FT-IR (KBr, cm^−1^): 405 (m), 420 (m), 474 (w), 490 (s), 496 (s), 608 (w), 729 (m), 752 (m), 764 (s), 791 (m), 812 (m), 827 (m), 854 (s), 868 (s), 995 (w), 1016 (w), 1053 (m), 1084 (m), 1094 (w), 1123 (m), 1138 (m), 1148 (m), 1175 (vs), 1217 (s), 1258 (s), 1308 (m), 1395 (m), 1410 (m), 1435 (vs), 1476 (vs), 1566 (s), 1595 (s), 2249 (vw), 2886 (w), 2920 (m), 3017 (w), 3053 (w), 3069 (w).

**[Cu_2_(Pic_3_PO)_2_I_2_]·2MeCN** (**3**·2MeCN). Off-white powder. Yield: 141 mg (82%). *Anal*. Calc. for C_40_H_42_I_2_Cu_2_N_8_O_2_P_2_: C, 43.3; H, 3.8; N, 10.1%. Found: C, 43.5; H, 3.9; N, 9.9%. FT-IR (KBr, cm^−1^): 405 (m), 420 (m), 473 (w), 488 (s), 496 (s), 608 (w), 727 (m), 764 (s), 789 (m), 799 (m), 812 (m), 827 (m), 854 (s), 868 (s), 995 (m), 1015 (w), 1055 (m), 1086 (m), 1123 (m), 1138 (m), 1150 (m), 1175 (vs), 1217 (s), 1258 (s), 1308 (m), 1404 (m), 1410 (m), 1435 (vs), 1474 (s), 1568 (s), 1597 (s), 2251 (w), 2880 (w), 2918 (m), 3019 (w), 3055 (w), 3071 (w).

**Complex 1:** The compound was obtained by desolvation of **1**·2MeCN by heating at 120 °C in vacuum (10^−2^ Torr) for 20 min. Off-white powder. *Anal*. Calc. for C_36_H_36_Cl_2_Cu_2_N_6_O_2_P_2_: C, 51.2; H, 4.3; N, 9.9%. Found: C, 51.0; H, 4.2; N 9.8%. FT-IR (KBr, cm^−1^): 420 (m), 490 (s), 758 (s), 787 (m), 827 (s), 856 (s), 995 (w), 1055 (w), 1080 (w), 1140 (m), 1175 (vs), 1219 (s), 1252 (s), 1312 (m), 1398 (m), 1437 (vs), 1476 (vs), 1566 (s), 1597 (s), 2891 (w), 2928 (w), 2949 (w), 3019 (w), 3053 (w).

### 3.3. X-ray Crystallography

Single crystals of **1**·2MeCN, **2**·2MeCN, and **3**·2MeCN were grown by slow evaporation of a MeCN solution of the corresponding compound. The diffraction data were collected on a Bruker Kappa Apex II CCD diffractometer using ϕ,ω-scans of narrow (0.5°) frames with MoKα radiation (λ = 0.71073 Å) and a graphite monochromator. The structures were solved by SHELXT 2014/5 [53] and refined by a full matrix least-squares anisotropic–isotropic (for H atoms) procedure using the SHELXL-2018/3 program set [54]. Absorption corrections were applied using the empirical multiscan method with the SADABS program [55]. The positions of the hydrogen atoms were calculated with the riding model.

CCDC 2232003 (for **1**·2MeCN), 2232004 (for **2**·2MeCN), and 2232005 (for **3**·2MeCN) contain the supplementary crystallographic data for this paper. These data can be obtained free of charge from The Cambridge Crystallographic Data Center at https://www.ccdc.cam.ac.uk/structures/ (accessed on 10 December 2022).

## 4. Conclusions

In conclusion, dinuclear complexes [Cu_2_X_2_(Pic_3_PO)_2_] (X = Cl, Br, I) have been designed exploiting tris(pyridin-2-ylmethyl)phosphine oxide (Pic_3_PO). The complexes are structurally characterized and investigated in terms of photophysics and quantum chemistry. At ambient temperature, the above compounds show cyan-to-green TADF of ^1^(M+X)LCT kind with quantum yields up to 54% and decay times of 4.3–9 µs. When passing from iodide (X = I) to chloride (X = Cl), an increase in the emission efficiency as well as a bathochromic shift of its maxima (λ_max_) are observed. In addition, X-ray radioluminescence is found for the obtained compounds. The similarity of emission profiles, recorded for the photo- and radioluminescence point to the similar radiative excited state for both processes. It is noteworthy that, compared to TADF, the radioluminescence efficiency grows in the order X = Cl < Br < I. The observed order is likely attributable to the increased absorption of X-rays by heavier atoms. The presented results provide new knowledge about the effect of halogens on photo- and radioluminescence of Cu(I) halide complexes.

## Figures and Tables

**Table 1:** Selected bond lengths (Å) in complexes **1**·2MeCN, **2**·2MeCN, **3**·2MeCN.

	1·2MeCN	2·2MeCN	3·2MeCN
Cu···Cu	3.1220(4)	3.2239(11)	3.2739(8)
Cu–N	2.0173(19), 2.030(2)	2.019(5), 2.025(6)	2.035(4), 2.048(4)
Cu–X (X = halide)	2.3471(7), 2.5916(8)	2.4894(11), 2.6973(14)	2.6612(7), 2.7919(8)

**Table 2:** Photoluminescence characteristics of synthesized complexes ^(a)^.

	1·2MeCN	1 ^(b)^	2·2MeCN	3·2MeCN
λ_max_ (298 K), nm	530	545	507	485
Φ_PL_ (298 K), %	54	35	44	30
τ (298 K), µs	4.3	5.2	9	9
λ_max_ (77 K), nm	535	547	514	492
τ (77 K), µs	25	26.6	34.5	48
∆E(S_1_–T_1_), cm^−1 (c)^	762	391	734	765

^(a)^ The data were recorded at λ_ex_ = 390 nm; ^(b)^ Desolvated complex **1** was obtained by heating **1**·2MeCN at 120 °C in vacuum; ^(c)^ Estimated by fitting τ(T) datasets with the equation for TADF model [17].

## Data Availability

Not applicable.

## Supplementary Materials

The supporting information can be downloaded at: https://www.mdpi.com/article/10.3390/ijms24065145/s1. References [56,57,58,59,60,61,62,63] are cited in the supplementary materials.
