# Supplementary material for: TADF and X-ray Radioluminescence of New Cu(I) Halide Complexes: Different Halide Effects on These Processes
Source: Int J Mol Sci. 2023 Mar 7;24(6):5145. doi: 10.3390/ijms24065145 (PMC10049412; doi:10.3390/ijms24065145)
Supplement: Supplementary file 1 [file ijms-24-05145-s001.zip › ijms-2211407-supplementary.pdf]

Supporting Information

TADF and X-ray radioluminescence of new Cu(I) halide complexes: different halide effects on these processes

Alexander V. Artem'ev,[1]* Andrey Yu. Baranov,[1] Alexey S. Berezin,[1] Dmitri V. Stass,[2,3] Christina Hettstedt,[4] Konstantin Karaghiosoff,[4] Irina Yu. Bagryanskaya[5]

[1] *Nikolaev Institute of Inorganic Chemistry, 3, Acad. Lavrentiev Ave., Novosibirsk 630090, Russia*
[2] *Novosibirsk State University, 2 Pirogova St., Novosibirsk 630090, Russia*
[3] *Voevodsky Institute of Chemical Kinetics and Combustion SB RAS, 3 Institutskaya St., 630090 Novosibirsk, Russia*
[4] *Department of Chemistry, Ludwig-Maximilian University of Munich, Butenandtstr. 5−13, 81377 Munich, Germany*
[5] *N. N. Vorozhtsov Novosibirsk Institute of Organic Chemistry, SB RAS, 9, Acad. Lavrentiev Ave., Novosibirsk 630090, Russia*

*Authors for correspondence: chemisufarm@yandex.ru

Table of contents

S2–4	§1. Single crystal X-ray crystallography
S4	§2. Powder X-ray diffraction data
S5	§3. TG&DTA curves
S5	§4. FT-IR spectra
S6–8	§5. Absorption, excitation and emission spectra
S8–12	§6. DFT computations
S12–14	§7. X-Ray radioluminescence
S14	§8. References

§1. Single crystal X-ray crystallography

Table S1. Data collection and selected refinement parameters for the obtained complexes.

	**1**·2MeCN	**2**·2MeCN	**3**·2MeCN
CCDC number	2232003	2232004	2232005
Chemical formula	$C_{36}H_{36}Cl_2Cu_2N_6O_2P_2 \cdot 2(C_2H_3N)$	$C_{36}H_{36}Br_2Cu_2N_6O_2P_2 \cdot 2(C_2H_3N)$	$C_{36}H_{36}Cu_2I_2N_6O_2P_2 \cdot 2(C_2H_3N)$
M_r	926.73	1015.65	1103.59
Crystal system, space group	Triclinic, $P\bar{1}$	Triclinic, $P\bar{1}$	Triclinic, $P\bar{1}$
Temperature (K)	296 K	296 K	296 K
a, *b*, *c* (Å)	9.8206 (7), 10.2907 (8), 10.6856 (9)	9.8727 (5), 10.4105 (7), 10.6724 (7)	9.9668 (10), 10.5931 (11), 10.7190 (12)
α, β, γ (°)	96.972 (4), 100.172 (4), 102.192 (4)	98.032 (3), 99.656 (2), 101.538 (2)	99.745 (5), 98.886 (5), 101.072 (4)
V (Å^3)	1024.72 (14)	1042.33 (11)	1074.0 (2)
Z	1	1	1
μ (mm^{-1})	1.29	3.06	2.55
Crystal size (mm)	0.30 × 0.15 × 0.06	0.45 × 0.25 × 0.08	0.50 × 0.10 × 0.02
T_{min}, T_{max}	0.815, 0.928	0.623, 0.862	0.631, 0.928
No. of measured, independent and observed [I> 2s(I)] reflections	20022, 5063, 3647	17523, 4071, 3726	22851, 5729, 4400
R_{int}	0.035	0.045	0.062
(sin θ/λ)$_{max}$ (Å^{-1})	0.671	0.617	0.707
$R[F^2$>2s(F^2)], $wR(F^2)$, S	0.038, 0.108, 1.03	0.059, 0.175, 1.05	0.055, 0.160, 1.05
No. of reflections	5063	4071	5729
No. of parameters	254	254	253
$\Delta\rho_{max}$, $\Delta\rho_{min}$ (e Å^{-3})	0.48, -0.35	1.34, -0.69	1.88, -1.88

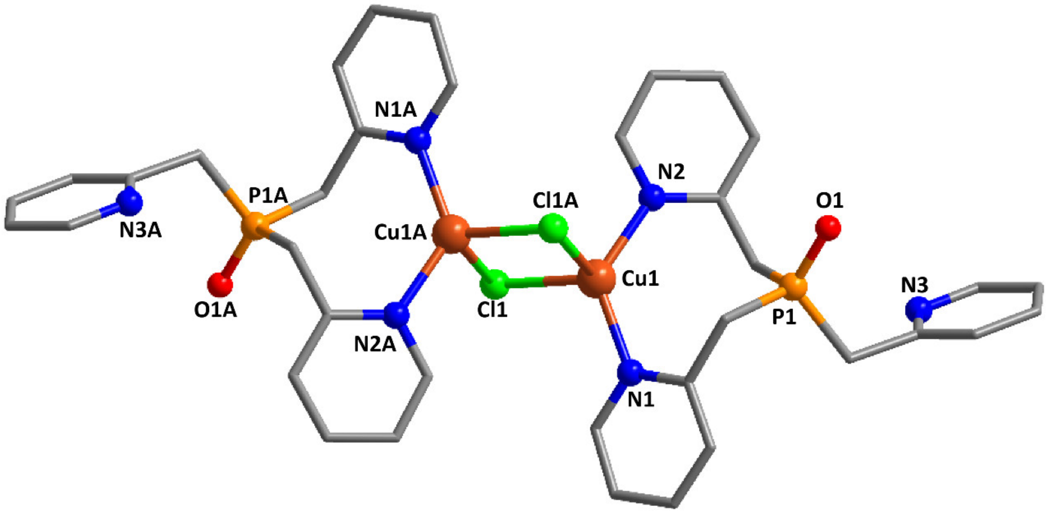

Figure S1. Structure of **1**·2MeCN according to single crystal XRD analysis (hydrogen atoms and solvent molecules are omitted for clarity). Selected bond length and angles: Cu1−Cu1A 3.1220(4), Cu1−N1 2.0173(19), Cu1−N2 2.030(2), Cu1−Cl1 2.3471(7), Cu1−Cl1A 2.5916(8), N1−Cu1−N2 128.01(7), N1−Cu1−Cl1 111.96(6), N2−Cu1−Cl1 108.38(6), N1−Cu1−Cl1A 103.09(6), N2−Cu1−Cl1A 99.29(6), Cl1−Cu1−Cl1A 101.76(2), Cu1−Cl1−Cu1A 78.24(2). Symmetry code: A(-x, -y, -z).

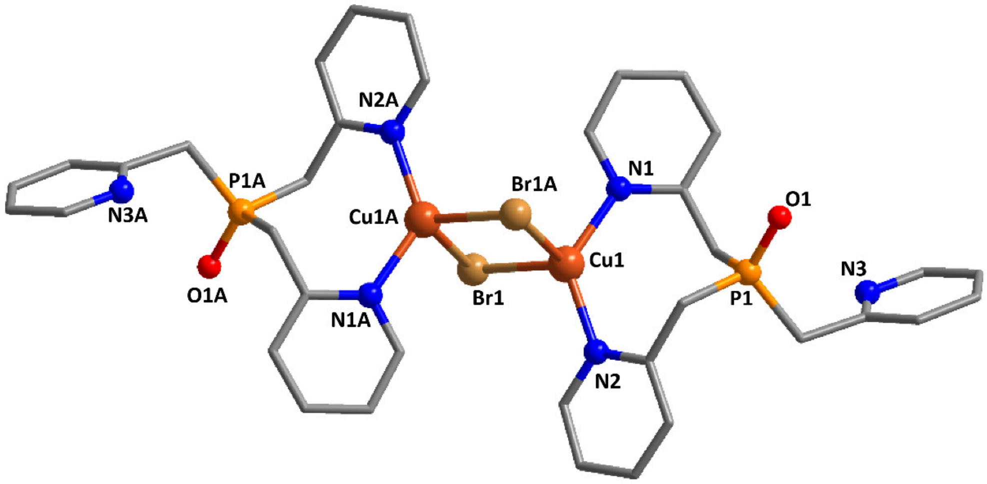

Figure S2. Structure of **2**·2MeCN according to single crystal XRD analysis (hydrogen atoms and solvent molecules are omitted for clarity). Selected bond length and angles: Cu1−Cu1A 3.2239(11), Br1−Cu1 2.4894(11), Br1−Cu1A 2.6973(14), Cu1−N2 2.019(5), Cu1−N1 2.025(6), Cu1−Br1−Cu1A 76.75(4), N2−Cu1−N1 128.6(2), N2−Cu1−Br1 111.92(15), N1−Cu1−Br1 105.57(17), N2−Cu1−Br1A 104.22(16), N1−Cu1−Br1A 99.90(17), Br1−Cu1−Br1A 103.25(4). Symmetry code: A(-1-x, 1-y, -z).

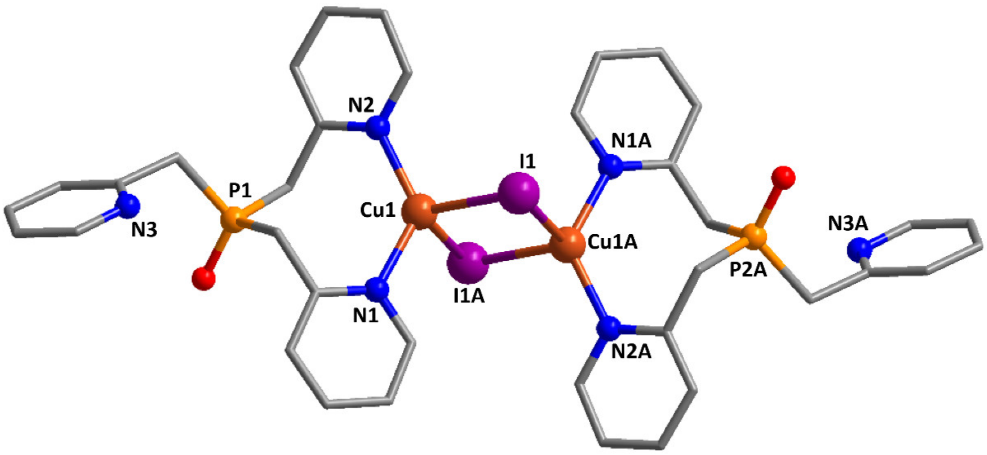

Figure S3. Structure of **3**·2MeCN according to single crystal XRD analysis (hydrogen atoms and solvent molecules are omitted for clarity). Selected bond length and angles: Cu1−Cu1A 3.2739(8), I1−Cu1 2.6612(7), I1−Cu1A 2.7919(8), Cu1−N2 2.035(4), Cu1−N1 2.048(4), Cu1−I1−Cu1A 73.75(2), N2−Cu1−N1 127.28(15), N2−Cu1−I1 111.59(11), N1−Cu1−I1 102.46(11), N2−Cu1−I1A 105.91(11), N1−Cu1−I1A 101.46(11), I1−Cu1−I1A 106.25(2). Symmetry code: A(2-x, 2-y, 2-z).

§2. Powder X-ray diffraction data

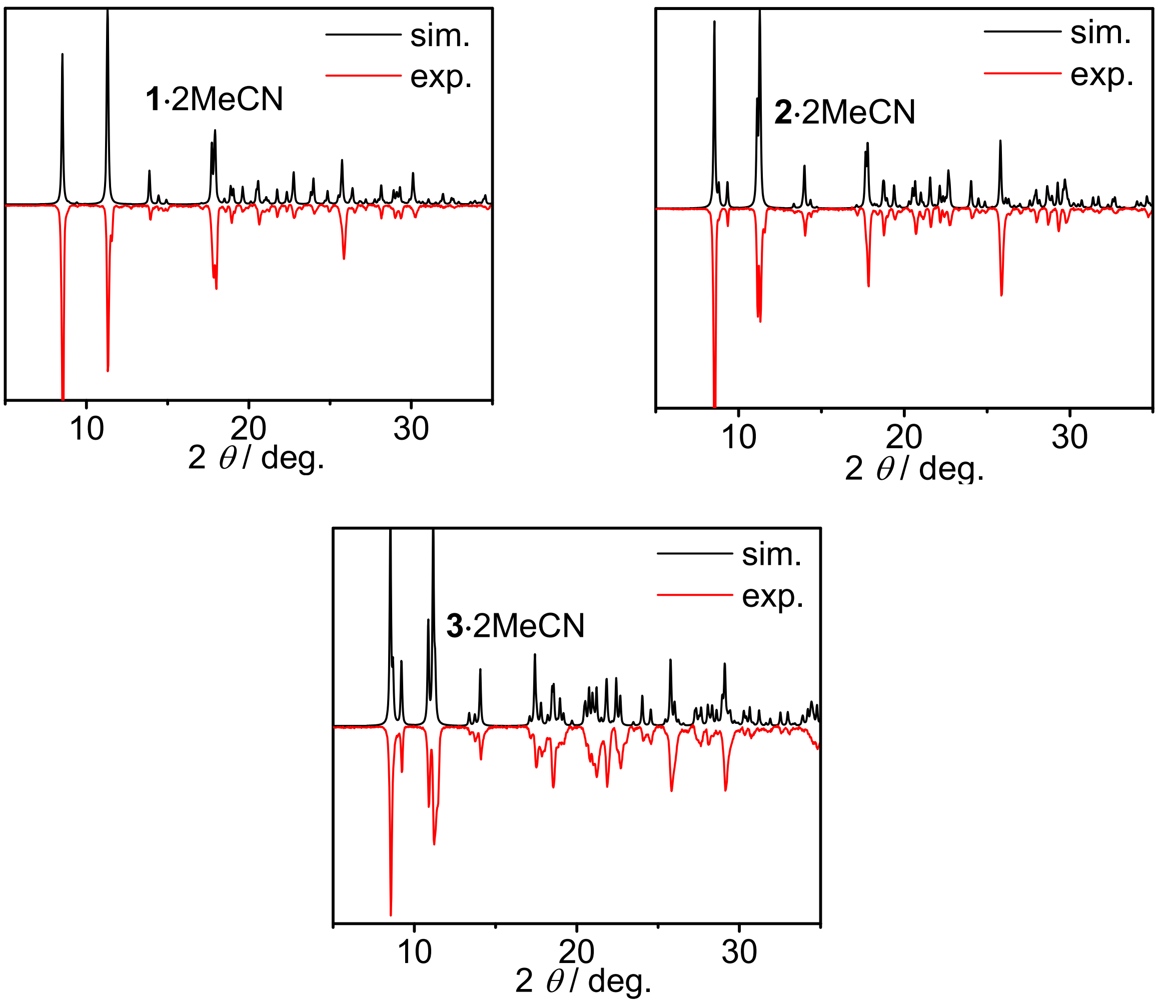

Figure S4. Experimental and simulated PXRD patterns of the synthesized compounds.

§3. TG&DTA curves

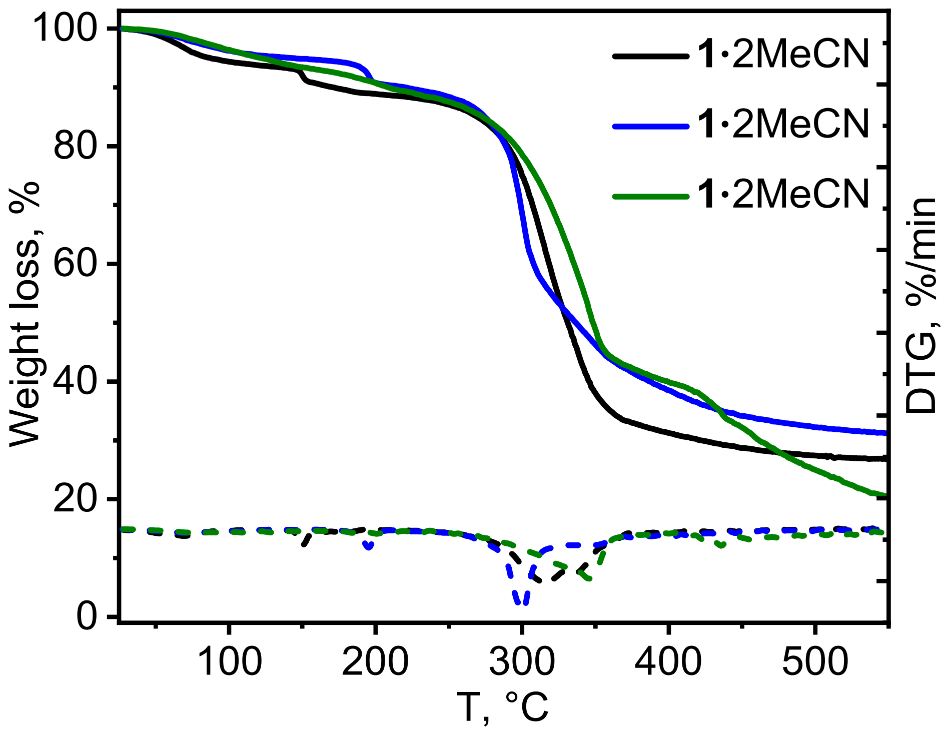

Figure S5. TGA and DTG curves for the synthesized compounds.

§4. FT-IR spectra

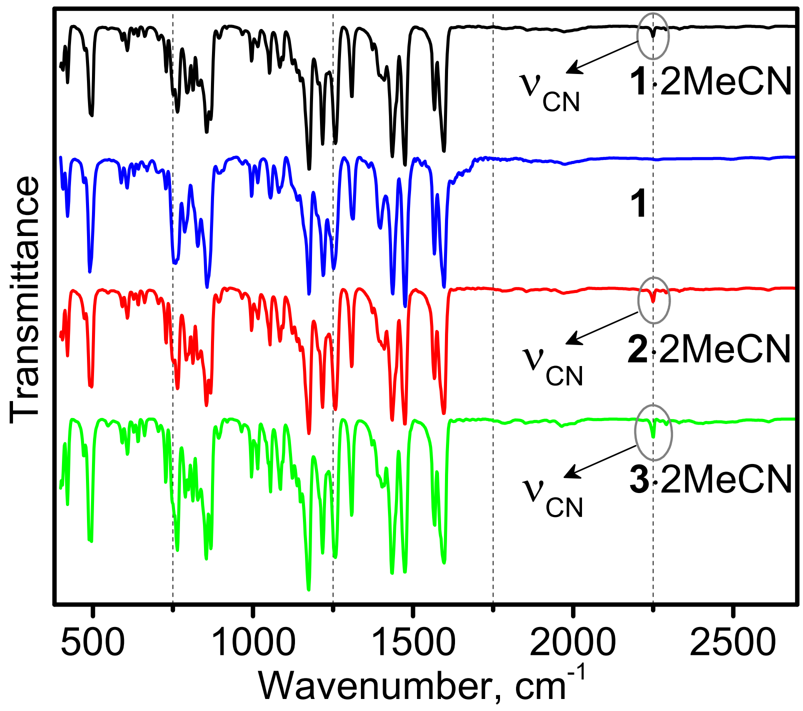

Figure S6. mid-IR spectra of the synthesized compounds.

§5. Absorption, excitation and emission spectra

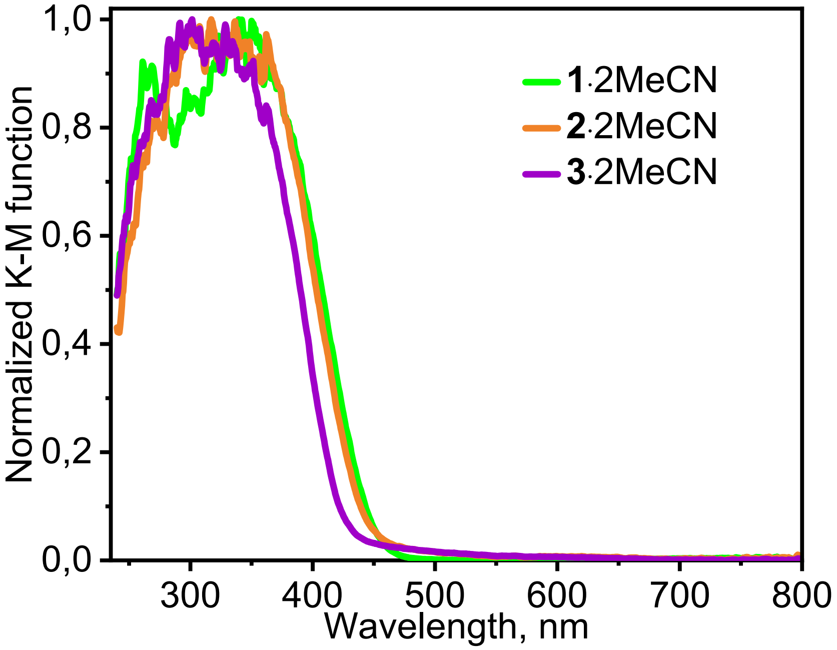

Figure S7. Absorption spectra (plotted as Kubelka-Munk function) of the samples.

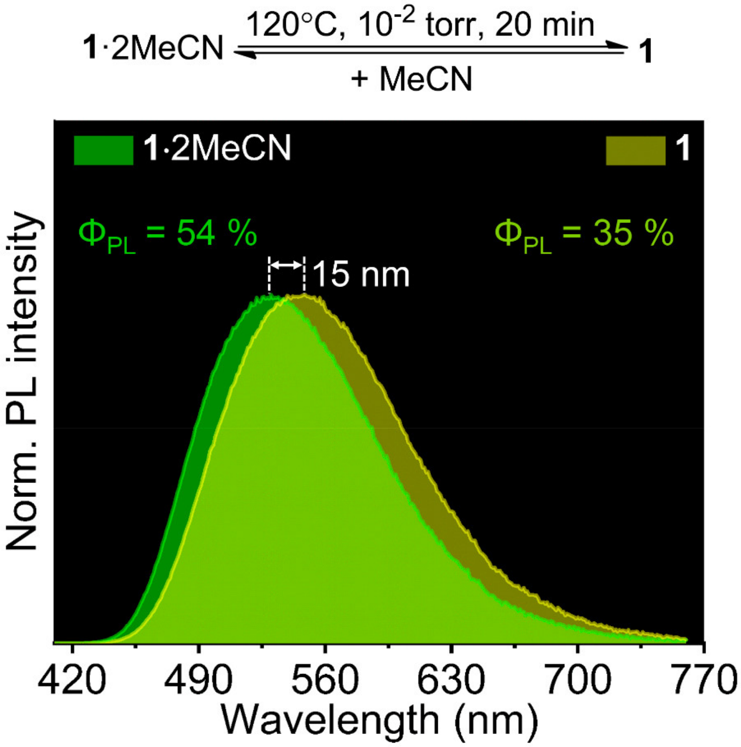

Figure S8. Evolution of solid-state emission properties upon desolvation of **1**·2MeCN.

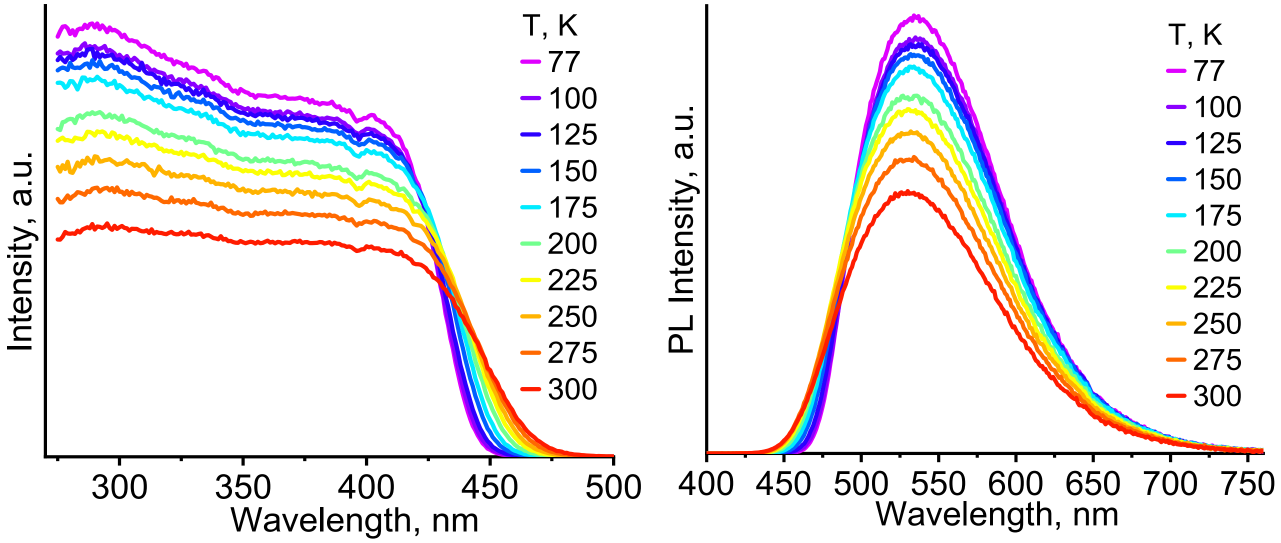

Figure S9. Temperature-dependent emission (left, λ_{ex} = 390 nm) and excitation (right, λ_{em} = 535 nm) spectra of **1**·2MeCN.

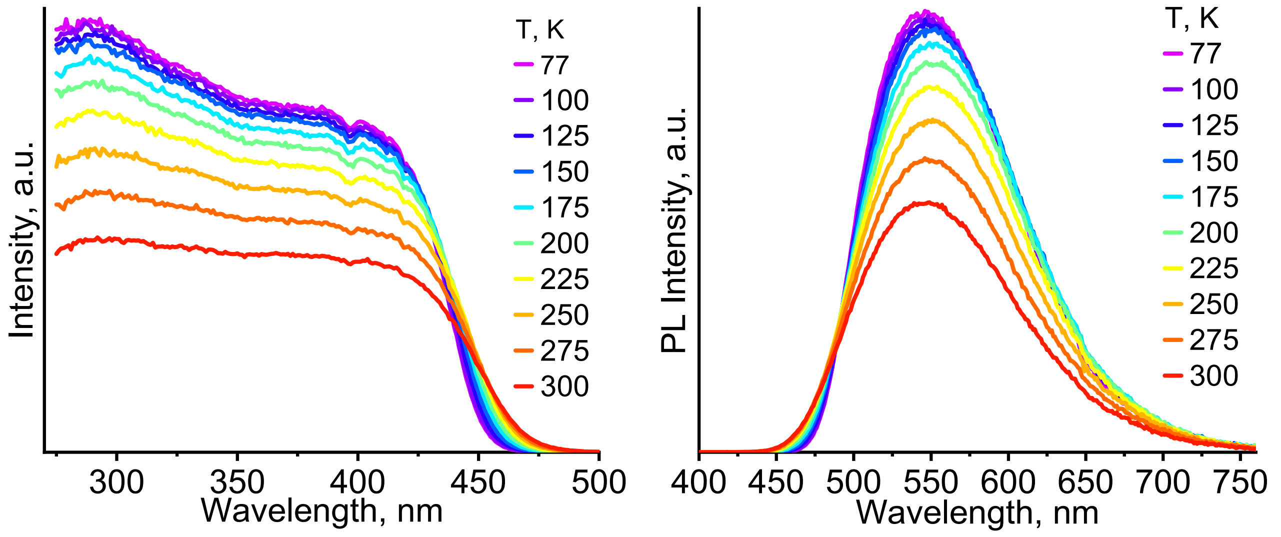

Figure S10. Temperature-dependent emission (left, λ_{ex} = 390 nm) and excitation (right, λ_{em} = 545 nm) spectra of **1**.

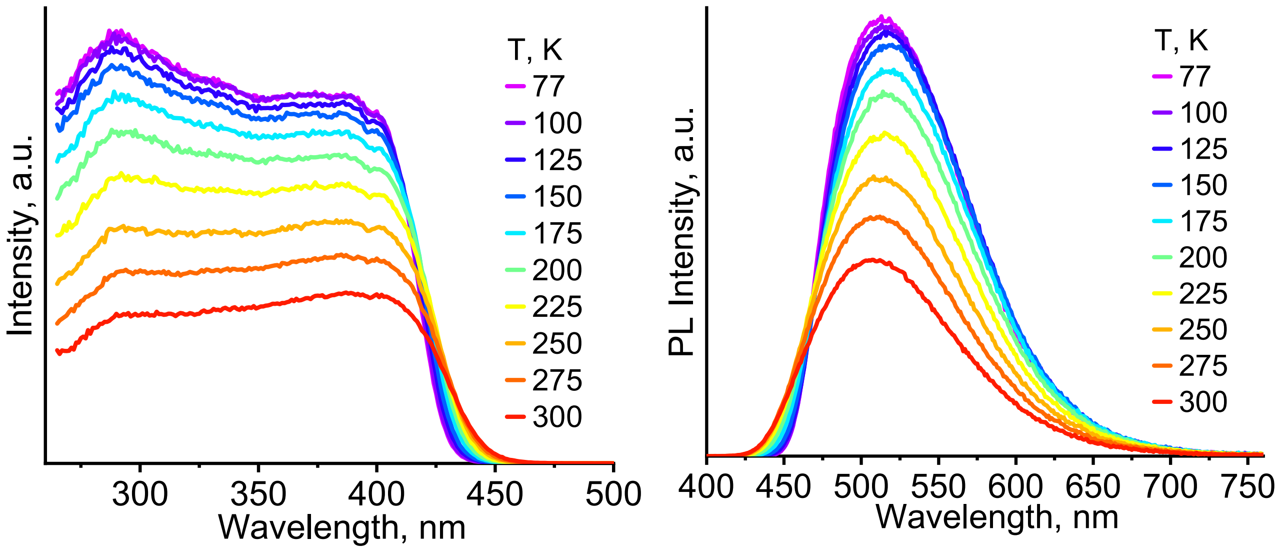

Figure S11. Temperature-dependent emission (left, λ_{ex} = 390 nm) and excitation (right, λ_{em} = 530 nm) spectra of **2**·2MeCN.

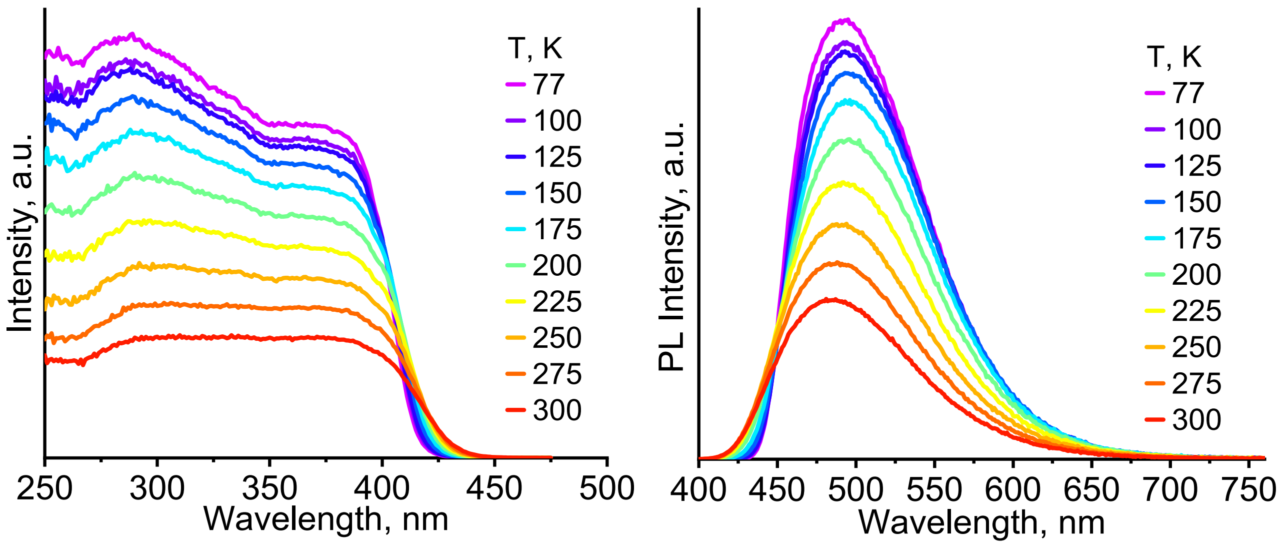

Figure S12. Temperature-dependent emission (left, λ_{ex} = 390 nm) and excitation (right, λ_{em} = 485 nm) spectra of **3**·2MeCN.

§6. DFT computations

DFT and TD-DFT computations of complex $[Cu_2Cl_2(Pic_3PO)_2]$ (**1**) were performed in gas phase using Gaussian 09 program [1]. The structures of the S_0 and T_1 states of **1** were fully optimized using B3LYP [2] hybrid functional coupled with the def2-TZVPP basis set [3]. No imaginary frequencies were found in all optimized structures. The absorption spectrum was computed using time-dependent DFT (TD-DFT) [4] calculations with the optimized ground state (S_0) geometry of **1**.

Table S2. Cartesian coordinates for gas phase S_0 and T_1 state geometries of **1** optimized at the B3LYP/def2TZVP level.

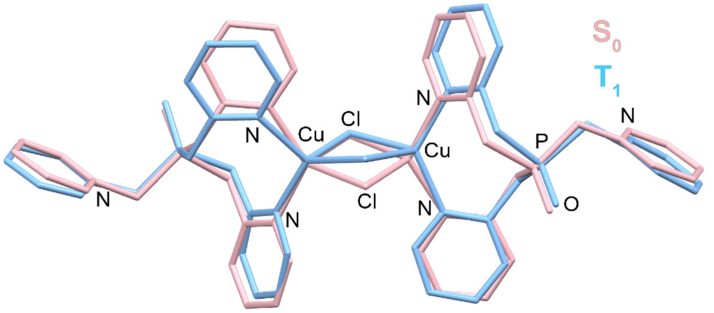

Ground state (S_0)				Lowest triplet excited state (T_1)			
Cu	0.854573000	0.825160000	0.947577000	Cu	-1.483430000	0.140457000	0.441863000
Cl	-0.827896000	-0.784675000	1.522356000	Cl	0.284179000	0.711654000	1.834905000
P	3.413837000	3.789221000	1.574176000	P	-5.290682000	-0.082764000	-1.043579000
N	4.153808000	4.935080000	4.885786000	N	-8.177311000	-0.778817000	0.811760000
N	0.061191000	2.714525000	1.458892000	N	-2.433753000	-1.527739000	0.311124000
O	4.707309000	3.842362000	0.848628000	O	-5.726704000	1.072631000	-1.872597000
N	2.797237000	0.265591000	1.455020000	N	-2.871278000	1.693918000	0.861748000
C	6.339858000	5.736762000	5.456696000	C	-10.345414000	0.117674000	0.316256000
C	-1.140548000	2.731632000	2.052851000	C	-2.072913000	-2.453107000	1.287263000
H	-1.524711000	1.762310000	2.346794000	H	-1.537759000	-2.056797000	2.140498000
C	-1.860523000	3.896604000	2.262283000	C	-2.362686000	-3.781945000	1.183786000
H	-2.825350000	3.854624000	2.747583000	H	-2.053434000	-4.441971000	1.984077000
C	-1.317000000	5.098239000	1.827326000	C	-3.067782000	-4.275886000	0.057833000
H	-1.852537000	6.029176000	1.958942000	H	-3.290918000	-5.324951000	-0.059224000
C	-0.072626000	5.085289000	1.217605000	C	-3.513544000	-3.318823000	-0.877896000
H	0.376037000	6.002943000	0.862627000	H	-4.114276000	-3.628764000	-1.724899000
C	0.605672000	3.876591000	1.053581000	C	-3.224955000	-1.983614000	-0.747644000
C	1.968505000	3.825513000	0.428102000	C	-3.790356000	-0.946569000	-1.665714000
H	2.143088000	4.702475000	-0.197487000	H	-4.083551000	-1.388007000	-2.619772000
H	2.055765000	2.944386000	-0.212375000	H	-3.063829000	-0.162786000	-1.895794000
C	3.226762000	2.330264000	2.679217000	C	-4.939115000	0.368475000	0.716265000
H	3.808741000	2.569896000	3.570044000	H	-5.912193000	0.432089000	1.203298000
H	2.181635000	2.244718000	2.975150000	H	-4.377201000	-0.443580000	1.171720000
C	3.701416000	1.039340000	2.079348000	C	-4.217964000	1.670551000	0.843067000
C	3.197308000	-0.912851000	0.956288000	C	-2.242635000	2.875461000	0.993442000
H	2.428233000	-1.502801000	0.478158000	H	-1.163752000	2.830883000	1.033686000
C	4.502707000	-1.364265000	1.036836000	C	-2.911320000	4.079754000	1.091565000
H	4.767966000	-2.325690000	0.620629000	H	-2.351941000	4.997913000	1.197349000
C	5.446880000	-0.553106000	1.652888000	C	-4.299434000	4.068814000	1.049313000
H	6.481575000	-0.861134000	1.723631000	H	-4.863830000	4.989656000	1.112641000
C	5.039557000	0.660323000	2.179692000	C	-4.950804000	2.856843000	0.927027000
H	5.744645000	1.322284000	2.661324000	H	-6.028672000	2.806478000	0.884243000
C	3.163361000	5.229230000	2.697301000	C	-6.576555000	-1.396352000	-0.898514000
H	3.055096000	6.095329000	2.041558000	H	-6.627081000	-1.863629000	-1.883506000
H	2.238700000	5.100446000	3.258282000	H	-6.239347000	-2.143658000	-0.181666000
C	4.308747000	5.428512000	3.652323000	C	-7.919852000	-0.855236000	-0.499544000
C	5.149097000	5.091188000	5.759746000	C	-9.361836000	-0.305294000	1.199559000
H	4.982065000	4.679766000	6.749123000	H	-9.530393000	-0.265513000	2.270326000
C	6.504761000	6.236092000	4.170582000	C	-10.072335000	0.052767000	-1.044549000
H	7.420613000	6.736857000	3.885337000	H	-10.804139000	0.380450000	-1.771339000
C	5.478459000	6.078889000	3.253105000	C	-8.844425000	-0.436195000	-1.459352000

H	5.579323000	6.434354000	2.237584000	H	-8.592446000	-0.486282000	-2.509208000
Cu	-0.854573000	-0.825160000	-0.947577000	Cu	1.685879000	0.185830000	-0.232780000
Cl	0.827896000	0.784675000	-1.522356000	Cl	-0.296869000	0.209533000	-1.607551000
P	-3.413837000	-3.789221000	-1.574176000	P	5.443472000	0.141429000	0.878398000
N	-4.153808000	-4.935080000	-4.885786000	N	8.007505000	-0.052068000	-1.659424000
N	-0.061191000	-2.714525000	-1.458892000	N	2.712704000	1.965293000	-0.495727000
O	-4.707309000	-3.842362000	-0.848628000	O	5.902627000	-0.655516000	2.042112000
N	-2.797237000	-0.265591000	-1.455020000	N	2.490250000	-1.684371000	0.004153000
C	-6.339858000	-5.736762000	-5.456696000	C	10.007080000	-1.311898000	-1.259186000
C	1.140548000	-2.731632000	-2.052851000	C	2.275908000	2.805354000	-1.445485000
H	1.524711000	-1.762310000	-2.346794000	H	1.465302000	2.434421000	-2.059186000
C	1.860523000	-3.896604000	-2.262283000	C	2.801560000	4.072871000	-1.630552000
H	2.825350000	-3.854624000	-2.747583000	H	2.410844000	4.709972000	-2.410917000
C	1.317000000	-5.098239000	-1.827326000	C	3.820845000	4.495308000	-0.787906000
H	1.852537000	-6.029176000	-1.958942000	H	4.252087000	5.482120000	-0.890890000
C	0.072626000	-5.085289000	-1.217605000	C	4.274357000	3.631132000	0.195758000
H	-0.376037000	-6.002943000	-0.862627000	H	5.059315000	3.934126000	0.874817000
C	-0.605672000	-3.876591000	-1.053581000	C	3.707408000	2.362560000	0.320168000
C	-1.968505000	-3.825513000	-0.428102000	C	4.182759000	1.399856000	1.368139000
H	-2.143088000	-4.702475000	0.197487000	H	4.649588000	1.932979000	2.197957000
H	-2.055765000	-2.944386000	0.212375000	H	3.341513000	0.838286000	1.778842000
C	-3.226762000	-2.330264000	-2.679217000	C	4.738578000	-0.864773000	-0.490843000
H	-3.808741000	-2.569896000	-3.570044000	H	5.601104000	-1.296136000	-1.000190000
H	-2.181635000	-2.244718000	-2.975150000	H	4.236811000	-0.202356000	-1.196278000
C	-3.701416000	-1.039340000	-2.079348000	C	3.808028000	-1.955058000	-0.042815000
C	-3.197308000	0.912851000	-0.956288000	C	1.642739000	-2.667523000	0.347664000
H	-2.428233000	1.502801000	-0.478158000	H	0.591476000	-2.418089000	0.349173000
C	-4.502707000	1.364265000	-1.036836000	C	2.063469000	-3.943028000	0.680203000
H	-4.767966000	2.325690000	-0.620629000	H	1.332373000	-4.693317000	0.943796000
C	-5.446880000	0.553106000	-1.652888000	C	3.423825000	-4.216586000	0.665907000
H	-6.481575000	0.861134000	-1.723631000	H	3.794996000	-5.197437000	0.931395000
C	-5.039557000	-0.660323000	-2.179692000	C	4.303111000	-3.211484000	0.297755000
H	-5.744645000	-1.322284000	-2.661324000	H	5.368946000	-3.386593000	0.275428000
C	-3.163361000	-5.229230000	-2.697301000	C	6.795292000	1.104651000	0.083409000
H	-3.055096000	-6.095329000	-2.041558000	H	7.117503000	1.825235000	0.838100000
H	-2.238700000	-5.100446000	-3.258282000	H	6.406342000	1.650156000	-0.775275000
C	-4.308747000	-5.428512000	-3.652323000	C	7.949055000	0.243164000	-0.356471000
C	-5.149097000	-5.091188000	-5.759746000	C	9.016188000	-0.810002000	-2.091716000
H	-4.982065000	-4.679766000	-6.749123000	H	9.029298000	-1.023267000	-3.154779000
C	-6.504761000	-6.236092000	-4.170582000	C	9.935540000	-1.016613000	0.096567000
H	-7.420613000	-6.736857000	-3.885337000	H	10.679928000	-1.395208000	0.784566000
C	-5.478459000	-6.078889000	-3.253105000	C	8.891708000	-0.230513000	0.558452000
H	-5.579323000	-6.434354000	-2.237584000	H	8.790547000	0.002629000	1.608823000
H	-7.112968000	-5.839812000	-6.205499000	H	10.806224000	-1.917959000	-1.662885000
H	7.112968000	5.839812000	6.205499000	H	-11.291903000	0.487608000	0.685578000

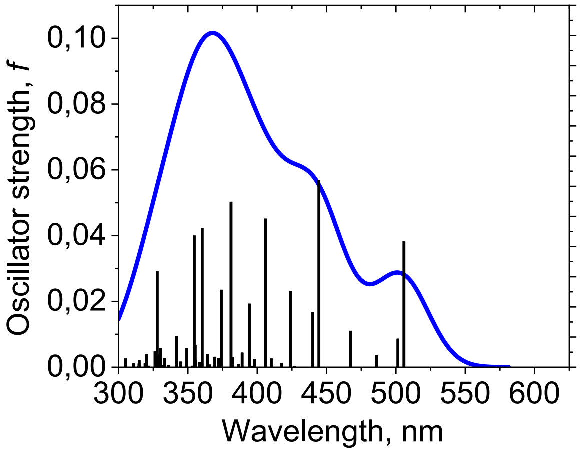

Figure S13. TD-TDF simulated absorption spectrum of **1**.

Table S3. Calculated (TD-B3LYP/def2TZVP) energies and characters of the main excitations (*f* > 0.002) of **1**.

E, eV	*f*	Transitions (main contributions)	Character
2.4514	0.0209	HOMO-1 -> LUMO (24.7%)<br>HOMO -> LUMO (64.6%)	(M+X)LCT
2.4729	0.0047	HOMO-1 -> LUMO (53.8%)<br>HOMO -> LUMO (29.3%)	(M+X)LCT
2.6534	0.006	HOMO -> LUMO+3 (66.7%)	(M+X)LCT
2.7897	0.0310	HOMO-1 -> LUMO+3 (64.4%)	(M+X)LCT
2.8177	0.0091	HOMO-3 -> LUMO+2 (22.9%)<br>HOMO-2 -> LUMO+3 (32.6%)	(M+X)LCT
2.9239	0.0126	HOMO-5 -> LUMO+1 (10.9%)<br>HOMO-4 -> LUMO+1 (64.2%)	(M+X)LCT
3.0546	0.0246	HOMO-5 -> LUMO+1 (58.1%)<br>HOMO-4 -> LUMO+1 (17.3%)	(M+X)LCT
3.1446	0.0105	HOMO-6 -> LUMO+1 (40.9%)<br>HOMO-4 -> LUMO+2 (29.9%)	(M+X)LCT
3.1865	0.0024	HOMO-3 -> LUMO+2 42.7%<br>HOMO-2 -> LUMO+3 34.1%	(M+X)LCT
3.2535	0.0274	HOMO-7 -> LUMO (61.4%)	(M+X)LCT
3.3145	0.0128	HOMO-6 -> LUMO+2 (34.1%)<br>HOMO-5 -> LUMO+2 (15.6%)	(M+X)LCT
3.4043	0.0021	HOMO-4 -> LUMO+6 (73.6%)	(M+X)LCT
3.4415	0.0023	HOMO-5 -> LUMO+2 (27.7%)<br>HOMO-6 -> LUMO+2 (25.1%)	(M+X)LCT
3.4902	0.0037	HOMO-7 -> LUMO+3 (32.6%)<br>HOMO -> LUMO+8 (19.9%)	(M+X)LCT
3.4966	0.0218	HOMO-8 -> LUMO (43.6%)<br>HOMO-5 -> LUMO+2 (23.2%)	(M+X)LCT
3.5509	0.0031	HOMO-5 -> LUMO+6 (68.0%)	(M+X)LCT
3.6259	0.0051	HOMO-9 -> LUMO+1 (76.9%)	(M+X)LCT

3.7526	0.0031	HOMO-5 -> LUMO+4 (60.1%)	(M+X)LCT
3.7815	0.0159	HOMO-8 -> LUMO+3 (52.3%)	(M+X)LCT
3.7998	0.0026	HOMO-6 -> LUMO+4 (71.1%)	(M+X)LCT

§7. X-Ray radioluminescence

X-ray radioluminescence (RL) spectra were recorded on a home-built spectrometer [5] following the earlier developed protocol for powder samples [6]; further technical details on possible experimental artifacts and spectral processing can be found in ref [7]. The sample of neat solid in the form of an island of finely ground powder with dimensions 3 x 8 mm and thickness of about 0.1 mm, applied via a stencil on a vertical aluminum plate with polypropylene-based double-sided Scotch tape, was directly exposed to the incident X-ray beam (unfiltered bremsstrahlung from a CW X-ray tube 2,5BSV-27-Mo, Svetlana, St. Petersburg, Russia, 40 kV x 20 mA, sample distance to anode 210 mm) and to the light-collecting optics of the detection system comprising a quartz optical imaging system, a grating monochromator (MDR-206, LOMO Photonics, St Petersburg, Russia, objective focus length 180 mm, grating 1200 lines per mm, inverse linear dispersion 4.3 nm mm^{-1}) with slits set to 2.2 mm/2.2 mm (spectral resolution about 10 nm), and a Hamamatsu H10493-012 photosensor module. All experiments were performed at ambient conditions in air without environmental control. To assess compound stability under X-ray irradiation, four consecutive spectra of single wavelength scans of 18 min each were recorded from freshly prepared samples, the gradual sagging of spectra is indicative of the degradation rate of unprotected neat powder under irradiation in air. The RL spectra given in the main text are averages of the four spectra. All RL spectra were recorded in nominally identical conditions and were normalized to sample amount in moles, the y-axes, although given in "arbitrary units", can be directly compared between different spectra.

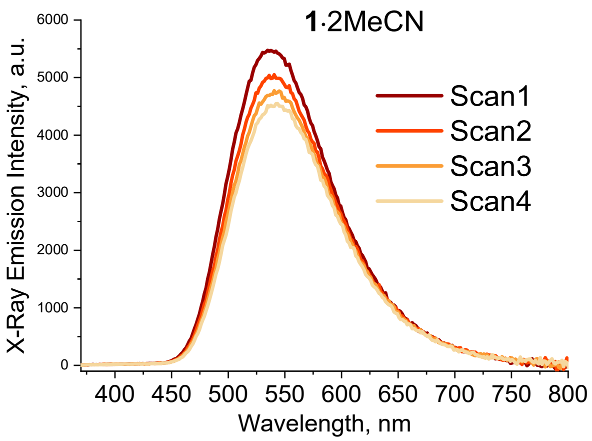

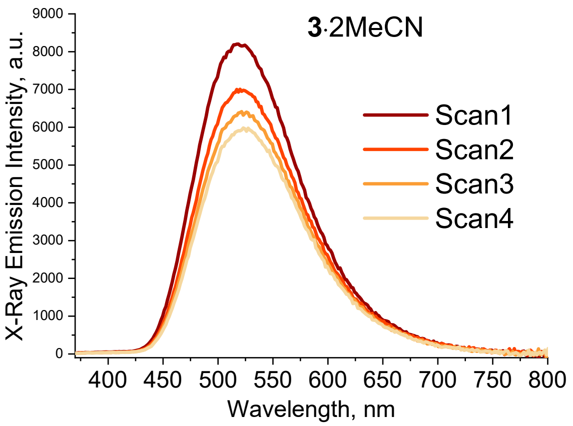

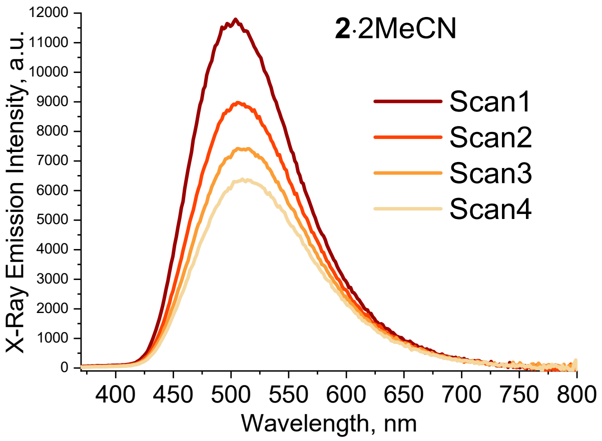

Figure S14. Comparison of stability of powders **1**–**3** toward X-rays (298 K).

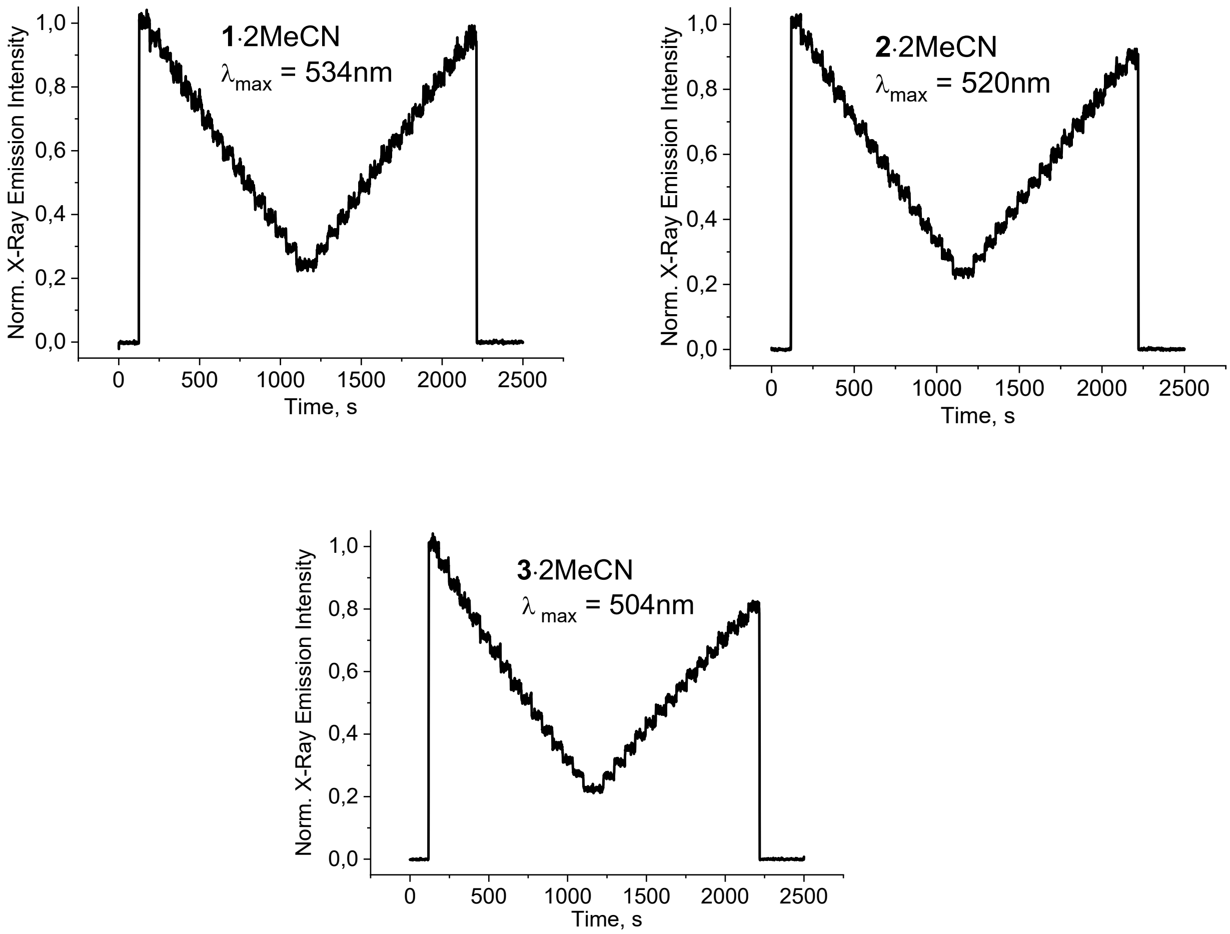

Figure S15. Check of linearity of RL response *vs.* dose rate for **1**–**3**. Detection wavelength was set at emission maximum, and emission intensity as raw signal from detector was recorded while varying anode current of the X-ray tube from nominal (20 mA) down to 5mA and back to 20 mA in 1 mA steps, recording signal for 1 min at each step (2 min at 5 mA). The accelerating voltage was held constant (40 kV), and thus anode current was directly proportional to dose rate, so the produced graph gives the dependence of emission intensity on

dose rate over 4:1 range, clearly demonstrating a linear dose rate response. Upon returning back to nominal anode current the signal does not return exactly to the initial level due to sample degradation, as shown in Fig. S14 above.

§8. References

1. M. Frisch, G.W. Trucks, H.B. Schlegel, G.E. Scuseria, M.A. Robb, J.R. Cheeseman, G. Scalmani, V. Barone, B. Mennucci, Ga. Petersson, others, Gaussian 09, revision D. 01, (2009).
2. P. J. Stephens, F. J. Devlin, C. F. Chabalowski and M. J. Frisch, *J. Chem. Phys.*, **1994**, *98*, 11623–11627.
3. B.P. Pritchard, D. Altarawy, B. Didier, T.D. Gibson, T.L. Windus, *J. Chem. Inf. Model.*, 2019, ***59***, 4814–4820.
4. (a) Bauernschmitt, R. Ahlrichs, *Chem. Phys. Lett.*, **1996**, *256*, 454–464; (b) C. Van Caillie, R.D. Amos, *Chem. Phys. Lett.*, **1999**, *308*, 249-255; (c) G. Scalmani, M.J. Frisch, B. Mennucci, J. Tomasi, R. Cammi, V. Barone, *J. Chem. Phys.*, **2006**, *124*, 094107.
5. E.V. Kalneus, A.R. Melnikov, V.V. Korolev, V.I. Ivannikov and D.V. Stass, *Appl. Magn. Reson.* **2013**, 44, 81–96.
6. D.V. Evtushok, A.R. Melnikov, N.A. Vorotnikova, Y.A. Vorotnikov, A.A. Ryadun, N.V. Kuratieva, K.V. Kozyr, N.R. Obedinskaya, E.I. Kretov, I.N. Novozhilov, Y.V. Mironov, D.V. Stass, O.A. Efremova and M.A. Shestopalov, *Dalton Trans.* **2017**, 46, 11738–11747.
7. D.V. Stass, N.A. Vorotnikova and M.A. Shestopalov, *J. Appl. Phys.* **2021**, 129, 183102.
